# Relative mortality in soft tissue sarcoma patients: a Danish population-based cohort study

**DOI:** 10.1186/1471-2407-14-682

**Published:** 2014-09-19

**Authors:** Katja Maretty-Nielsen, Ninna Aggerholm-Pedersen, Johnny Keller, Akmal Safwat, Steen Baerentzen, Alma B Pedersen

**Affiliations:** Sarcoma Centre of Aarhus University Hospital, Aarhus, Denmark; Department of Experimental Clinical Oncology, Aarhus University Hospital, Noerrebrogade 44, building 5, 8000 Aarhus C, Denmark; Department of Oncology, Aarhus University Hospital, Noerrebrogade 44, 8000 Aarhus C, Denmark; Department of Orthopaedic Surgery, Aarhus University Hospital, Noerrebrogade 44, 8000 Aarhus C, Denmark; Department of Pathology, Aarhus University Hospital, Noerrebrogade 44, 8000 Aarhus C, Denmark; Department of Clinical Epidemiology, Aarhus University Hospital, Olof Palmes Allé 43-45, 8200 Aarhus N, Denmark

**Keywords:** Soft tissue sarcoma, Relative survival, Prognosis, Cancer-specific survival, Comorbidity

## Abstract

**Background:**

Cancer-specific survival estimates rely on precise and correct data on the cause of death; however, these data can be difficult to acquire, particularly in elderly patients where comorbidity is common. Furthermore, while some deaths are directly related to cancer, others are more complex, with cancer merely contributing. Another, more precise, method is to assess the relative mortality, i.e., mortality in patients compared to the general population. The aims of this study were to describe the relative mortality in soft tissue sarcoma, and to compare the relative mortality with the cancer-specific mortality.

**Methods:**

We included 1246 patients treated for soft tissue sarcoma and 6230 individually age- and sex-matched individuals from the general population. The relative mortality was estimated as rates, and rate ratios adjusted for comorbidity. Mortality rate ratios were computed using the Cox proportional hazard model for 0–5 years and 5–10 years, according to age, sex and level of comorbidity. The cancer-specific mortality was compared to the corresponding relative mortality.

**Results:**

The overall 5- and 10-year relative mortality was 32.8% and 36.0%. Patients with low-grade soft tissue sarcoma did not have increased mortality compared with the general population. Soft tissue sarcoma patients had a 4.4 times higher risk of dying within the first five years after diagnosis and a 1.6 times higher risk between five and ten years compared with the general comparison cohort. The relative mortality varied according to age, grade, stage at diagnosis, and level of comorbidity, being highest in younger patients and in patients without comorbidity. The overall 5- and 10-year cancer-specific mortality was underestimated by 1.5 and overestimated by 0.7 percentage points compared to the relative mortality, respectively. No statistical significant difference between the relative and the cancer-specific mortality was found.

**Conclusion:**

The relative mortality provides an unbiased and accurate method to differentiate between cancer-specific and non-cancer-specific deaths. However, when data on the cause of death is of a sufficient quality, there is no difference between relative mortality and disease-specific mortality based on death certificates.

**Electronic supplementary material:**

The online version of this article (doi:10.1186/1471-2407-14-682) contains supplementary material, which is available to authorized users.

## Background

The mortality among soft tissue sarcoma patients has been studied numerous times [1–7]. While some studies report mortality as overall, most use cancer-specific measures, since this is expected to better reflect the “true” mortality caused by the sarcoma [2–7]. However, using cancer-specific measures entails two potential problems; misclassification of the underlying cause of death, and no consensus on which causes of death are related to the cancer.

Assessing cancer-specific mortality relies on precise and correct data on the cause of death; however, these data can be difficult to achieve, particularly in elderly patients where comorbidity is common. An autopsy remains the best method to determine the cause of death; however, the autopsy rate in Denmark has declined rapidly, as observed in most countries [8–10]. The cause of death is therefore often registered by physicians, either the deceased’s general practitioner or hospital doctors, and the validity of the registered cause of death is thus dependent on the physicians’ knowledge of preceding diseases. Previous studies have concluded that causes of death are associated with issues of inaccuracy and substantial variability of coding according to cancer type, age at death and time period [8, 11–16]. Furthermore, while some deaths are directly related to cancer, others are more complex, with cancer merely contributing to the death [17]. In these cases, assigning death as either cancer-specific or not can be problematic and ambiguous.

Another method to obtain the “true” mortality caused by the cancer is by assessing the relative mortality, i.e., the mortality in cancer patients compared with the mortality in a general population without cancer [18]. The mortality in the general population can be determined using life tables or randomly selected individual age- and sex-matched controls, a unique possibility in Denmark.

Cancer-specific estimates based on death certificates have been compared to relative estimates in other cancers and disease types, with varying results; however, the correlation between the relative and the disease-specific mortality has, to our knowledge, not previously been investigated in soft tissue sarcoma [19, 20].

The aims of this study were therefore to estimate the relative mortality in patients with soft tissue sarcoma using an age- and sex-matched general comparison cohort, as well as to compare the relative mortality with the cancer-specific mortality based on death certificates.

## Methods

### Setting

This cohort study was conducted in western Denmark within a population of approximately 2.5 million [21]. The public health care system in Denmark is tax-funded and free of charge, allowing free access to hospital care for all citizens. All residents in Denmark are assigned a unique 10-digit number, the CPR number, which is used throughout Danish society, including the health care system. This allows for linkage on an individual level between registries.

### Data sources

The Aarhus Sarcoma Registry is a population-based registry including all sarcoma patients in western Denmark treated at the Aarhus Sarcoma Centre from 1st January 1979 to 31th December 2008. The registry has previously been systematically validated, and contains basic patient data including date of diagnosis, and detailed data on tumour characteristics, treatment, and follow-up [22].

The Danish Civil Registration System holds information on all residents in Denmark since 1968 and is updated on a daily basis. The registry encompasses both historical and current data, including CPR number, municipality of residence, vital status, as well as date of birth, emigration, and/or death [23].

The Danish National Patient Registry has collected data on all non-psychiatric admissions to Danish hospitals since 1977, including visits to hospital outpatient clinics and emergency rooms since 1995. For each medical contact the CPR number, date of admission and discharge, as well as up to 20 discharge diagnoses is recorded. The discharge diagnoses are coded by physicians according to the eighth (before 1994) and tenth version of the International Classification of Disease (ICD-8 and ICD-10), and include both main and secondary diagnoses [24–27].

The Danish Cause of Death Registry contains data on the immediate and underlying cause of death, according to the ICD-8 and ICD-10, based on the diagnosis from the death certificates. The completion of death certificates for any death occurring in Denmark is mandatory, and data has been registered since 1875 [28].

### Soft tissue sarcoma patients

The Aarhus Sarcoma Registry was used to identify all soft tissue sarcoma patients in western Denmark diagnosed between 1979 and 2008 (N = 1753). In this study we focused on patients with tumours located in the extremities or trunk wall, and therefore 455 patients with tumours located, e.g., retroperitoneally, intraabdominally, or in the head and neck, were excluded. Furthermore, patients (N = 52) with specific histological subtypes traditionally not considered as a classical soft tissue sarcoma, e.g., gastrointestinal stromal tumours, kaposis sarcoma, atypical fibroxanthoma, and atypical lipomatous tumours were excluded, leaving 1246 patients for the analysis.

Patients at the Aarhus Sarcoma Centre are diagnosed and treated by an experienced multidisciplinary team, according to international and national guidelines [29, 30]. Sarcomas were classified using the grading system described by Jensen et al. [31]. In general, most patients were treated with surgery, with the aim of a wide margin, followed by radiotherapy for deep intermediate and high-grade tumours [32].

### General comparison cohort

A random general comparison cohort was sampled from the general population by individual matching using the Civil Registration System [23]. For each soft tissue sarcoma patient registered in the Aarhus Sarcoma Registry we identified 5 age- and sex-matched individuals from the general population, who were alive at the date of sarcoma diagnosis (index date), had not previously been diagnosed with a sarcoma, and lived in the same geographical area as the soft tissue sarcoma patient (the same county).

### Comorbidity

Data on comorbidity in both the soft tissue sarcoma patients and the general comparison cohort was obtained by individual linkage (CPR number) with the National Patient Registry [24, 27]. All discharge diagnoses between 1st January 1977 and the date of diagnosis (index date) were retrieved. We excluded all discharge diagnoses within 30 days, and all cancer diagnoses within 90 days prior to the date of diagnosis in the soft tissue sarcoma patients, to eliminate nonspecific symptoms or hospitals admissions related to the sarcoma. Comorbidity was assessed using the Charlson Comorbidity Index [33]. The ICD codes used to determine the Charlson Comorbidity Index score are shown in an additional table [See Additional file 1: Table S1].

### Statistical analyses

**Figure 1:**
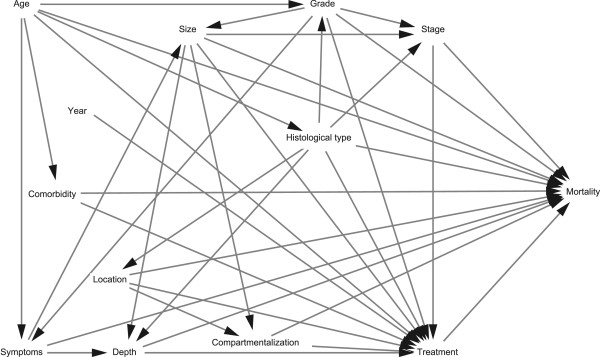
**Directed acyclic graph of the possible relationship between important covariates and mortality in soft tissue sarcoma patients.**

Baseline characteristics were summarized as medians and interquartile ranges for continuous variables, and numbers and percentages for categorical variables. The prevalence of comorbidity in the soft tissue sarcoma patients and the general comparison cohort was compared using the chi-squared test. All individuals were followed from index date to date of death, emigration, or end of the study (15th July 2013). Data on death were obtained from the Civil Registration System. The main outcome measure assessed was relative mortality, computed as one minus the relative survival (S_r_), where the relative survival [18] is defined as the ratio of the observed overall survival of soft tissue sarcoma patients (S_o_) and the observed survival in the age- and sex matched general comparison cohort (S_e_):


The relative mortality was estimated as relative mortality rates (RMRs) and mortality rate ratios (MRRs). The overall mortality was estimated for soft tissue sarcoma patients and the general comparison cohort separately, using the Kaplan-Meier method [34], and the 10-year RMRs with 95% confidence intervals (CIs) were computed. RMRs were computed both as overall and according to histological grade and subtypes. MRRs were estimated as hazard ratios, using a Cox proportional hazard model, adjusting for age, sex, and level of comorbidity [35]. Age and comorbidity were included, as seen in Table 1. Age, sex, comorbidity, and time-specific estimates were computed. To estimate the impact of treatment, stage-specific MRRs were computed using a model adjusting for age, comorbidity, compartmentalization, depth, grade, histological type, location, and size. The adjustment covariates were selected based on a modified version (Figure 1) of a directed acyclic graph constructed by Maretty-Nielsen et al. [36, 37] and included as seen in Tables 1 and 2. The proportional hazard assumption was assessed graphically using log-log plots. Based on this it was found that the assumption was not met and MRRs were therefore analysed separately from 0–5 years and from 5–10 years. No violation of the proportional hazard assumption was found within these follow-up periods.

**Table 1:** **Overall mortality and mortality rate ratios at diagnosis/index date for soft tissue sarcoma patients (N = 1246) and the general comparison cohort (N = 6230)**

		STS patient mortality % (95% CI)	General comparison mortality % (95% CI)	Crude MRR (95% CI)	Adjusted MRR (95% CI)*
**0 to 5 years**					
**Gender**	Female	40.2 (36.4-44.3)	10.7 (9.7-11.9)	4.8 (4.1-5.7)	4.7 (3.9-5.6)
	Male	41.6 (37.9-45.5)	13.5 (12.4-14.7)	3.9 (3.4-4.6)	4.2 (3.6-4.9)
**Age (years)**	0-39	27.2 (22.5-32.7)	0.3 (0.1-0.7)	116.7 (42.7-318.6)	110.8 (40.5-303.0)
	40-59	24.4 (20.3-29.2)	2.4 (1.8-3.3)	11.5 (8.0-16.4)	11.0 (7.6-15.8)
	60-79	53.9 (49.5-58.5)	16.5 (15.1-18.1)	4.7 (4.0-5.5)	4.4 (3.7-5.1)
	≥ 80	72.2 (64.3-79.6)	51.2 (47.4-55.1)	1.9 (1.5-2.4)	1.9 (1.5-2.4)
**Comorbidity**	None	34.9 (31.9-38.0)	7.0 (6.3-7.8)	6.2 (5.4-7.2)	6.6 (5.7-7.7)
	Low	52.3 (43.3-62.0)	24.3 (21.2-27.8)	2.9 (2.1-3.9)	3.0 (2.2-4.1)
	Moderate	60.6 (51.5-69.7)	34.2 (29.7-39.2)	2.5 (1.9-3.4)	2.8 (2.1-3.8)
	High	68.7 (58.8-78.2)	48.9 (43.0-55.3)	1.8 (1.3-2.4)	2.0 (1.4-2.7)
**Total**		41.0 (38.3-43.7)	12.2 (11.4-13.0)	4.3 (3.8-4.8)	4.4 (3.9-4.9)
**5 to 10 years**					
**Gender**	Female	16.0 (12.3-20.6)	11.7 (10.4-13.1)	1.4 (1.1-2.0)	1.8 (1.3-2.5)
	Male	19.2 (15.4-23.9)	15.2 (13.8-16.7)	1.3 (1.0-1.7)	1.5 (1.2-2.0)
**Age (years)**	0-39	8.1 (5.1-13.0)	0.8 (0.4-1.4)	11.1 (5.1-24.6)	11.1 (5.0-24.6)
	40-59	12.4 (8.8-17.3)	4.4 (3.5-5.6)	3.1 (2.0-4.8)	2.9 (1.8-4.4)
	60-79	28.4 (22.3-35.8)	24.3 (22.2-26.5)	1.2 (0.9-1.6)	1.2 (0.9-1.6)
	≥ 80	60.0 (42.8-77.7)	64.0 (57.9-70.0)	1.0 (0.6-1.7)	1.1 (0.7-1.7)
**Comorbidity**	None	13.7 (11.1-16.9)	11.1 (10.1-12.2)	1.4 (1.1-1.8)	1.5 (1.2-2.0)
	Low	41.3 (26.5-60.4)	23.0 (18.8-27.9)	1.3 (0.8-2.3)	1.6 (0.9-2.8)
	Moderate	44.9 (30.6-62.3)	23.9 (19.9-28.6)	1.9 (1.1-3.3)	2.8 (1.6-4.9)
	High	32.9 (17.8-55.7)	20.1 (15.6-25.6)	0.7 (0.3-1.5)	0.9 (0.4-2.0)
**Total**		17.7 (14.9-20.9)	13.5 (12.5-14.5)	1.4 (1.1-1.7)	1.6 (1.3-2.0)

NOTES: *Abbreviations:* *STS* soft tissue sarcoma, *MRR* mortality rate ratio, *CI* confidence interval. *Adjusted for age, gender, and level of comorbidity.

**Table 2:** **Clinico-pathological characteristics of soft tissue sarcoma patient (N = 1246)**

		N	%
**Age (years)**	Median (interquartile range)	58	(41–71)
**Gender**	Female	587	47.1
	Male	659	52.9
**Stage at diagnosis**	Localized	1098	88.1
	Metastatic	148	11.9
**Location**	Upper extremity	190	15,3
	Trunk	447	35.9
	Lower extremity	603	48.4
	Disseminated/unknown	6	0.5
**Depth**	Subcutaneus	374	30.0
	Subfascial	872	70.0
**Tumour size (cm)** ^**a**^	< 5	410	33.7
	5-9	394	32.4
	≥ 10	411	33.8
**Histological grade**	Low	223	17.9
	Intermediate	168	13.5
	High	855	68.6
**Treatment**	Surg	773	62.0
	Surg + Rt	299	24.0
	Surg + Ch	45	3.6
	Surg + Rt + Ch	48	3.9
	Rt	17	1.4
	Ch	14	1.1
	Rt + Ch	20	1.6
	None	30	2.4

NOTES: *Abbreviations:* *Surg* surgery, *Rt* radiotherapy, *Ch* chemotherapy. ^a^31 missing values.

The cancer-specific mortality included all deaths from sarcoma or deaths with metastatic sarcoma. A death was considered as cancer-specific if the medical files rendered the death likely to be a consequence of the soft tissue sarcoma, e.g., death of a patient with multiple lung metastases and evident pneumonia. Data on the cause of death were retrieved from the Aarhus Sarcoma Registry and the Danish Cause of Death Registry. The cancer-specific mortality rate was estimated using the Kaplan-Meier method. The 5- and 10-year cancer-specific mortality was compared to the corresponding RMR for the entire soft tissue sarcoma cohort as well as according to stage at diagnosis.

All tests were two-sided and a *p*-value ≤ 0.05 was considered significant. Analyses were performed using the statistical software Stata, version 11.2.

### Ethics

The study was approved by the Danish Data Protection Agency, the Danish Health and Medicines Authority, and the National Committee on Health Research Ethics.

## Results

### Descriptive data

We identified 1246 soft tissue sarcoma patients and 6230 general comparison cohort individuals. The soft tissue sarcoma patient characteristics are shown in Table 2. The most frequent histological types of soft tissue sarcoma were malignant fibrous histiocytoma (300 [24.1%]), liposarcoma (195 [15.7%]), and leiomyosarcoma (190 [15.3%]). The prevalence of comorbidity in soft tissue sarcoma patients and the general comparison cohort is shown in Table 3. The prevalence of the various medical conditions was comparable, except for ‘any tumour’ and ‘metastatic solid tumour’. The prevalence of ‘Any tumour’ was 1.9 times (95% CI: 1.5-2.3) higher and ‘metastatic solid tumour’ was 7.3 times (95% CI: 4.2-12.5) higher in the soft tissue sarcoma patients, compared to the general comparison cohort. The median follow up period was 6.6 years (interquartile range 1.7-13.7) in soft tissue sarcoma patients and 11.2 years (interquartile range 6.8-17.7) in the general comparison cohort.

**Table 3:** **Comorbidity in soft tissue sarcoma patients (N = 1246) and the general comparison cohort (N = 6230) before diagnosis/index date according to the Charlson Comorbidity Index**

Condition	STS patients N (%)	General population cohort N (%)	P-value ^†^
Myocardial infarct	44 (3.5)	188 (3.0)	0.34
Congestive heart failure	22 (1.8)	133 (1.8)	0.91
Peripheral vascular disease	27 (2.2)	100 (1.6)	0.16
Cerebrovascular disease	44 (3.5)	259 (4.2)	0.31
Dementia	5 (0.4)	39 (0.6)	0.34
Chronic pulmonary disease	47 (3.8)	259 (4.2)	0.53
Connective tissue disease	18 (1.4)	97 (1.6)	0.77
Ulcer disease	37 (3.0)	167 (2.7)	0.57
Mild liver disease	7 (0.6)	31 (0.5)	0.77
Diabetes	33 (2.7)	164 (2.6)	0.97
Hemiplegia	1 (0.1)	4 (0.1)	0.84
Moderate/severe renal disease	11 (0.9)	39 (0.6)	0.31
Diabetes with end organ damage	7 (0.6)	67 (1.1)	0.10
Any tumour*	121 (9.7)	323 (5.2)	<0.001
Leukemia	4 (0.3)	9 (0.1)	0.17
Lymphoma	5 (0.4)	14 (0.2)	0.26
Moderate/severe liver disease	0 (0.0)	6 (0.1)	0.27
Metastatic solid tumour	32 (2.6)	22 (0.4)	<0.001
AIDS	0 (0.0)	3 (0.1)	0.44

NOTES: *Abbreviations:* *STS* soft tissue sarcoma, *AIDS* acquired immunedeficiency syndrome. ^†^Chi-squared test. *Excluding tumour in soft tissue and bone (ICD-8; 170, 171, 192.49-99 and ICD-10; C40-C41, C47, C49).

### Overall mortality

**Figure 2:**
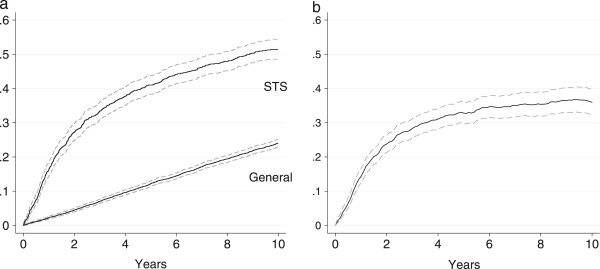
**Overall mortality for soft tissue sarcoma (STS) patients and the general comparison cohort (a) and relative mortality for soft tissue sarcoma patients (b) with 95% confidence interval.**

In total, 735 (59.0%) of the soft tissue sarcoma patients and 2265 (36.4%) of the general comparison cohort died during the follow-up period. The overall mortality for the soft tissue sarcoma patients and the general comparison cohort is shown in Figure 2. The mortality for soft tissue sarcoma patients was higher than that of the general comparison cohort during the entire follow-up period. The 5- and 10-year overall mortality were 41.0% (95% CI: 38.3-43.8) and 51.4% (95% CI: 48.6-54.3) for soft tissue sarcoma patients compared with 12.2% (95% CI: 11.4-13.0) and 24.0% (95% CI: 22.9-25.2) for the general comparison cohort, respectively. 70% of the observed deaths in soft tissue sarcoma patients within five years and 53% within ten years were related to the soft tissue sarcoma.

### Relative mortality

**Figure 3:**
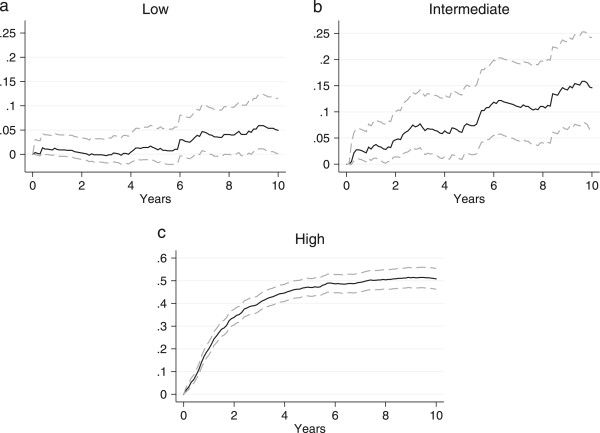
**Relative mortality for soft tissue sarcoma patients according to histological grade (a: low, b: intermediate, c: high) with 95% confidence interval.**

**Figure 4:**
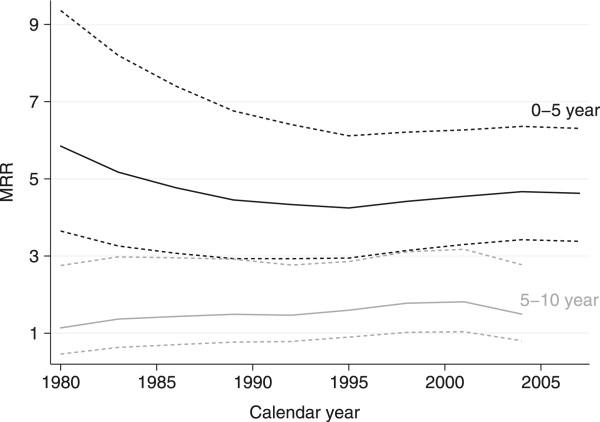
**Temporal trends in 0–5 year (black lines) and 5–10 year (grey lines) mortality rate ratios (MRR) with 95% confidence intervals (dotted) of 1246 soft tissue sarcoma patients diagnosed at the Aarhus Sarcoma Centre between 1979 and 2008 adjusted for age, sex, and level of comorbidity.**

The overall RMR are shown in Figure 2b. The mortality was not significantly increased in patients with low grade soft tissue sarcoma compared to the general comparison cohort, as seen in Figure 3a. The mortality was increased in patients with intermediate and high grade soft tissue sarcoma; however, while the increase in mortality was constant for intermediate soft tissue sarcoma, the majority of deaths occurred within the first five years for high grade soft tissue sarcoma (Figure 3b and c). The 5-year RMR according to histological subtypes is shown in Table 4. The 5- and 10-year MRR according to sex, age, and level of comorbidity are shown in Table 1. The overall risk of dying was 4.4 times (95% CI: 3.9-4.9) higher for the soft tissue sarcoma patients within the first five years after diagnosis, compared with the general comparison cohort, while ‘only’ 1.6 times (95% CI: 1.3-2.0) higher within the subsequent five years. The higher mortality was observed within all age and sex groups, except for soft tissue sarcoma patients ≥ 60 years, where the risk of dying five to ten years after diagnosis was equal to that of the general comparison cohort. The adjusted MRRs were higher in patients without comorbidity within the first five years and decreased with an increased level of comorbidity. The temporal trend in excess mortality for soft tissue sarcoma patients is shown in Figure 4. A tendency towards an increased 0–5 year MRR was observed in the first third of the study period, however neither the 0–5 nor the 5–10 year MRR changed significantly over the study period. The 5-year MRR according to treatment regimens in patients with localized and metastatic disease is shown in Table 5. The lowest excess mortality was observed in soft tissue sarcoma patients with localized disease treated solely with a wide resection, whereas the highest was observed in patients with untreated metastatic disease.

**Table 4:** **5-year relative mortality according to histological subtypes (N = 1246)**

	N	RM, %	95% CI
MFH	300	28.0	21.4-35.2
Liposarcoma	195	18.1	11.3-26.1
Leiomyosarcoma	190	33.4	25.6-41.9
Dermatofibrosarcoma	97	2.1	0.0-9.1
Synovial sarcoma	79	46.4	35.4-58.5
MPNST	78	52.4	40.8-64.5
Fibrosarcoma	53	30.3	18.1-46.0
Rhabdomyosarcoma	35	79.3	64.3-90.9
Angiosarcoma	32	56.8	18.1-75.5
Extraosseous osteosarcoma	25	48.6	27.6-71.4
PNET	25	59.7	41.4-78.5
Epithelioid sarcoma	21	27.9	13.2-52.4
Malignant hemangiopericytoma	20	40.9	21.0-66.3
Extraosseous chondrosarcoma	12	37.5	14.2-71.1
Unclassifiable	48	52.3	36.1-68.8
Others	36	31.5	17.3-50.6

NOTES: *Abbreviations:* *RM* relative mortality, *CI* confidence interval, *MFH* malignant fibrous histiocytoma, *MPNST* malignant peripheral nervesheath tumour, *PNET* primitive neuroectodermal tumour. Classified according to the WHO Classification of tumours of soft tissue and bone 3rd edition, 2002.

**Table 5:** **Adjusted mortality rate ratios according to treatment-regimes in patients with localized and metastatic soft tissue sarcoma (N = 1246)**

	N	5-year adjusted MRR (95% CI)*
**Localized** ^**†**^		
Wide -Rt	595	3.1 (2.6-3.5)
Wide + Rt	157	4.4 (3.0-6.7)
Intra -Rt	125	3.5 (2.5-4.9)
Intra + Rt	127	4.3 (3.0-6.0)
Rt	12	15.3 (5.4-43.4)
Ch +/−Surg/Rt	57	190.4 (48.6-745.3)
None	15	12.4 (4.7-32.2)
Total	1098	3.8 (3.3-4.3)
**Metastatic**		
Surg/Rt	63	15.7 (10.0-24.7)
Ch +/−Surg/Rt	70	228.4 (85.7-608.7)
None	15	236.1 (32.44-1717.9)
Total	148	25.3 (18.2-35.1)

NOTES: *Abbreviations:* *MRR* mortality rate ratio, *CI* confidence interval, *Wide* wide resection, *Rt* radiotherapy, *Intra* marginal/intralesional resection, *Ch* chemotherapy, *Surg* surgery. *Adjusted for age, level of comorbidity, compartmentalization, depth, grade, type, location and size. ^†^Margin missing in 10 cases.

### Relative vs. cancer-specific mortality

**Figure 5:**
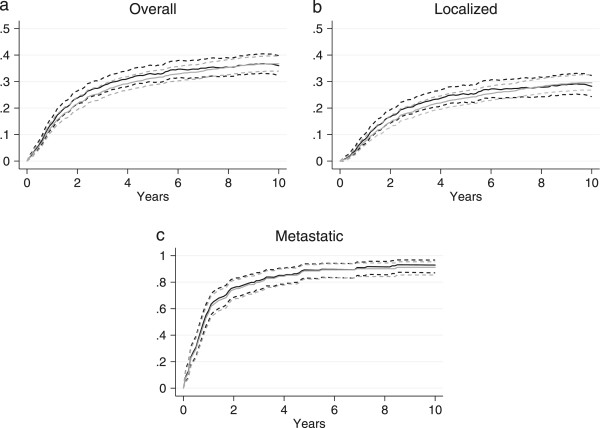
**Relative (black lines) and cancer-specific mortality (grey lines) for soft tissue patients overall (a) as well as stratified by stage at diagnosis (b-c) with 95% confidence interval.**

The relative and the cancer-specific mortality curves are depicted in Figure 5. The 5- and 10-year relative mortality for the entire soft tissue sarcoma cohort was 32.8% (95% CI: 29.8-36.0) and 36.0% (95% CI: 32.3-39.8), compared to a cancer-specific mortality of 31.3% (95% CI: 28.7-34.1) and 36.7% 95% (CI: 33.9-39.7), respectively. There was no statistically significant difference between the 5- and 10-year cancer-specific mortality and the relative mortality (5-year: 1.5 percentage point (pp) [−2.7-5.7], p = 0.24, 10-year: −0.7 pp [−5.5-4.1], p = 0.61). The 5- and 10-year relative mortality in soft tissue sarcoma patients with localized disease at diagnosis was 25.2% (95% CI: 22.1-28.5) and 28.2% (95% CI: 24.3-32.3), while the corresponding cancer-specific mortality was 23.8% (95% CI: 21.3-26.5) and 29.6% 95% (CI: 26.8-32.6), respectively. The 5- and 10-year cancer-specific mortality were 1.4 (95% CI: −2.8-5.7, p = 0.26) higher and 1.3 pp (95% CI: −3.7-6.4, p = 0.70) lower than the relative mortality in soft tissue sarcoma patient with localized disease, respectively. In soft tissue sarcoma patients with metastatic disease at diagnosis the 5-year relative- and cancer-specific mortality was 89.1% (95% CI 83.0-93.8) and 88.5% (95% CI 82.3-93.2), increasing to 92.9% (95% CI 87.0-96.7) and 91.2% (95% CI 85.4-95.4) at 10 years, respectively. No statistically significant discrepancy between the relative and the cancer-specific mortality was observed in patients with metastatic disease (5-year: 0.7 pp [−6.3-7.6], p = 0.42, 10-year: 1.7 pp [−4.5-7.8], p = 0.28). The shape of the relative mortality curve was similar to the cancer-specific mortality curve both in the entire cohort as well as in the localized and metastatic cohort, with the majority of sarcoma deaths occurring within the first 5 years of follow up (Figure 5).

## Discussion

To our knowledge, this is the first study of relative mortality in soft tissue sarcoma patients using individual age- and sex-matched comparison cohort from the general population. In this population-based cohort study we found that soft tissue sarcoma was associated with a higher mortality than the general comparison cohort, with an overall 5- and 10-year relative mortality of 32.8% and 36.0%. Soft tissue sarcoma patients had a 4.4 times higher risk of dying within the first five years after diagnosis and a 1.6 times higher risk between five and ten years, compared with the general comparison cohort. No statistically significant discrepancy between the relative and the cancer-specific mortality was found.

### Relative mortality

The relative mortality rates in sarcoma according to histological and anatomical subtypes have recently been investigated using national life tables [38, 39]. Results from the RARECARE project showed an overall 5-year relative mortality of 42% in soft tissue sarcoma, when including all anatomical locations. In comparison, our study population including only tumours located in the extremity or trunk wall reported a 5-year relative mortality of 32.0% for soft tissue sarcomas in the limbs and 55.9% for STSs in the superficial trunk [38]. Ng et al. reported the 5-year relative mortality according to histological type, grade and stage, based on data from the Surveillance, Epidemiology, and End Results (SEER) database [39]. The 5-year relative mortality varied from 5% to 15% in low-grade malignant fibrous histiocytoma (MFH), liposarcoma and leiomyosarcoma, i.e., the three most frequent subtypes in our study, compared to our result of 1.2%. Patients with localized MFH, liposarcoma, and leiomyosarcoma had 5-year relative mortalities of 20%, 24%, and 10%, respectively, while the corresponding mortalities in patients with metastatic disease were 89%, 88%, and 74%, respectively [39].

The adjusted MRRs according to age, sex and comorbidity level have not previously been reported in soft tissue sarcoma; however, our results are in accordance with findings from other studies [40, 41]. Within the first five years since diagnosis, soft tissue sarcoma patient had higher mortality than the corresponding population comparison cohort, independently of age, sex, and level of comorbidity. However if soft tissue sarcoma patients survived beyond the first five years after their diagnosis their risk of dying compared to the general comparison cohort varied, with no additional risk for patients ≥60 years and patients with severe comorbidity. This is in overall consistency with previous reports showing that 85-90% of relapses occur within the first five years and that patients cured of soft tissue sarcoma, i.e., patients who do not experience relapses, generally do not have significantly increased mortality compared to the general population [2, 42–44]. A recent study investigated the long term survival in soft tissue sarcoma patients who were alive and event-free more than five years after primary treatment, and reported 6.1% relapses beyond the first five years, with an overall survival of 97% [45].

### Relative vs. cancer-specific mortality

To our knowledge, the correlation between the relative and the disease-specific mortality in soft tissue sarcoma patients has not previously been investigated. The relative mortality in our study was similar to the estimated cancer-specific mortality, with discrepancies less than 2 pp in both the overall cohort as well as in patients with localized and metastatic disease.

The correlation between relative and cancer-specific mortality has been investigated in other cancers, with varying results depending on the type of cancer [19, 20, 46–51]. A study of 2777 breast cancer patients reported a 4 pp lower cancer-specific than relative mortality at both 5 and 10 years, while a study of 25,531 gastroinstinal carcinoid patients reported a 3.9 pp higher cancer-specific mortality [19, 20]. A study comparing relative and cancer-specific survival in rectal cancer patients, using national life tables to adjust for baseline mortality, reported a 10-year relative survival of 66.5% (95% CI 61.3-72.1) and a 10-year cancer-specific survival of 66.4% (95% CI 62.5-70.5), supporting that the relative mortality in general is an unbiased, precise estimate for the cancer-specific survival [48].

### Methodological considerations

The major strengths of this study include the large number of soft tissue sarcoma patients and the use of population-based, systematically validated data from the Aarhus Sarcoma Registry [22]. Furthermore, the use of the Danish health care registries allows for virtually complete follow up, thus limiting the risk of selection bias.

Our study had some limitations. The information on comorbidity was extracted from an administrative registry, where coding errors may be expected to some extent. However since data in the National Patient Registry is registered prospectively and independently of this study, any misclassifications are expected to be non-differential.

The relative mortality method relies on internal comparability between the cohort of interest (soft tissue sarcoma patients) and the general comparison cohort, and violation of this can result in possible bias [52]. Mortality in the general population can be assessed using either national life tables or a matched general comparison cohort. In either case, the general population from which the data is acquired is assumed to be free of the disease of interest, with all excess mortality being due to the disease; however, when using life tables this is rarely the case. Although, since soft tissue sarcoma is a very rare disease, the issue of soft tissue sarcoma patients being included in the data on which the life tables are derived is considered minor. The internal comparability in this study is thus expected to be better than studies using national life-tables, since patients were matched individually by age, sex, and geographical region, and patients with previously diagnosed sarcoma were excluded. Furthermore, since comorbidity is closely related to mortality, data on previous comorbidity was obtained for both soft tissue sarcoma patients and general population individuals and included in the analysis. A high internal comparability is also supported by the similar distribution of the medical conditions used in the Charlson Comorbidity Index. Soft tissue sarcoma patients had a larger prevalence of ‘Any tumour’ and ‘Metastatic solid tumour’; however, this might be explained by the aetiology of some sarcomas, being induced by previous treatment with radio- or chemotherapy. Some studies have reported differences in survival according to the social economic status in other cancer types; however, this has not been investigated in soft tissue sarcoma. We did not have data on social economic status, but since the study was conducted in Denmark where all inhabitants have free access to health care, we expect any possible confounding due to this to be minor. However, when using a matched general comparison cohort to assess the mortality in the general population there is a potential sampling bias, which is not present when using life tables.

The cancer-specific method relies on identifying the ‘true’ underlying cause of death, as misclassification of this can result in possible bias. Even in cases where the correct immediate cause of death is known, the contribution of the cancer as part of the underlying cause can be impossible to determine, rendering the distinction between cancer-specific and non-cancer-specific deaths meaningless [52]. The impact of the disease, e.g., on public health, is therefore expected to be better captured using the relative mortality method than the cancer-specific method.

## Conclusion

In this population-based cohort study of soft tissue sarcoma patients with tumours located in the extremity or trunk wall, the overall 5- and 10-year relative mortality was 32.8 and 36.0%. Patients with low grade soft tissue sarcoma did not have increased mortality compared with the general population. The relative mortality varied according to age, grade, stage of diagnosis, and level of comorbidity, being highest in younger patients and in patients without comorbidity. The relative mortality provides an accurate, unbiased method to differentiate between cancer-specific and non-cancer-specific deaths. However, when data on the cause of death is of a sufficient quality, there is no difference between relative mortality and disease-specific mortality based on death certificates.

## Electronic supplementary material

Additional file 1: Table 1: The ICD-8 and ICD-10 codes used to calculate the Charlson Comorbidity Index and the matching scores for the 19 medical conditions. (XLSX 12 KB)
